# N/S-Co-Doped Porous Carbon Sheets Derived from Bagasse as High-Performance Anode Materials for Sodium-Ion Batteries

**DOI:** 10.3390/nano9091203

**Published:** 2019-08-27

**Authors:** Lili Wang, Lei Hu, Wei Yang, Dewei Liang, Lingli Liu, Sheng Liang, Caoyu Yang, Zezhong Fang, Qiang Dong, Chonghai Deng

**Affiliations:** Department of Chemical and Materials Engineering, Hefei University, Hefei 230601, China

**Keywords:** sodium-ion batteries, N/S co-doped, carbon, anode materials, porous, bagasse

## Abstract

Heteroatom doping is considered to be an efficient strategy to improve the electrochemical performance of carbon-based anode materials for Na-ion batteries (SIBs), due to the introduction of an unbalanced electron atmosphere and increased electrochemical reactive sites of carbon. However, developing green and low-cost approaches to synthesize heteroatom dual-doped carbon with an appropriate porous structure, is still challenging. Here, N/S-co-doped porous carbon sheets, with a main pore size, in the range 1.8–10 nm, has been fabricated through a simple thermal treatment method, using KOH-treated waste bagasse, as a carbon source, and thiourea, as the N and S precursor. The N/S-co-doped carbon sheet electrodes possess significant defects, high specific surface area, enhanced electronic conductivity, improved sodium storage capacity, and long-term cyclability, thereby delivering a high capacity of 223 mA h g^−1^ at 0.2 A g^−1^ after 500 cycles and retaining 155 mA h g^−1^ at 1 A g^−1^ for 2000 cycles. This work provides a low-cost route to fabricate high-performance dual-doped porous carbonaceous anode materials for SIBs.

## 1. Introduction

With the development of the global economy, the use of clean and renewable energy sources, such as wind, solar, geothermal, and hydropower has spurred significant worldwide benefits. To achieve this goal, efficient electric energy storage (EES) technologies are needed to allow these intermittent renewable energy sources to provide continuous power. Advanced EES systems are primarily based on battery technologies. Lithium-ion batteries (LIBs), which have become dominant power supplies for consumer electronics and electric vehicles (EVs), represent one of the most mature commercial technologies in the field. However, the increasing cost of lithium metal has constrained further applications of LIBs [1,2]. In recent years, as an alternative to LIBs, room temperature sodium ion batteries (SIBs) have offered great potential for large-scale energy storage requirements, due to their low cost and rich abundance in sodium resources. However, as the most conventional anodes for LIBs, graphite is not an appropriate material for SIB, due to its insufficient interlayer distance, leading a slower kinetics of Na^+^ insertion/extraction. Therefore, there is an urgent need to develop high-performance SIB anode materials that enable fast Na^+^ storage kinetics.

The anode materials for SIBs, such as carbonaceous materials [1,2,3], oxides [4], sulfides [5,6], phosphides [7], alloys [8], and Ti-based intercalation compounds [9], have been extensively investigated. Among them, carbonaceous materials, especially the disordered carbon, have gained a great deal of attention as promising candidates because of their high electronic conductivity, eco-friendliness, and thermal stability [10]. However, the specific capacity of non-graphitic carbon materials, is still far from that achieved by graphite in a lithium system. Recently, great efforts have been invested to fabricate carbon nanostructures for better electrochemical performance, such as porous carbon fibers [11], hollow carbon nanospheres [12,13], and carbon nanosheets [14,15,16,17]. Among them, two-dimensional (2D) porous carbon materials have been regarded as potential structures for Na^+^ storage. With the unique properties of 2D materials, porous carbon sheets can promote rapid ion transport and enable large surface-to-volume ratios for sodium storage, thus enabling great improvements in power and energy density, compared to bulk electrodes [14,15,16,17].

Another promising approach is the introduction of heteroatoms (nitrogen, boron, sulfur, and phosphorous), since doping the covalent heteroatom is capable of turning the surface chemistry and electronic structure properties of carbonaceous material [18]. The introduction of nitrogen is beneficial for improving the electronic conductivity and the electron-donating ability of the carbonaceous materials by generating extrinsic defects [2]. Furthermore, the heteroatom of sulfur is also proposed to enhance electrode kinetics by forming a –C–S–C– covalent bond and providing additional active sites for Na^+^, as well as enhancing the reversible capacity by affecting the interlayering of carbon [19,20,21]. For example, Zhang et al. prepared S-doped mesoporous carbon, derived from the pyrolysis treatment of MOF-5 and inorganic sulfur powders, exhibiting a reversible capacity of 173.7 mA h g^−1^ at 0.2 A g^−1^ [22]. Furthermore, the co-doping of nitrogen and sulfur synergically facilitate electron transport and improve Na^+^ storage capacities [21]. For example, S/N-co-doped carbon has been developed by using citric acid and urea as carbon and nitrogen source, and H_2_S gas as sulfur source, thereby exhibiting a high capacity of 211 mA h g^−1^ at 1 A g^−1^ after 1000 cycles, which is much higher than that of merely N-doped carbon [23]. Hence, constructing N/S-co-doped carbon materials would achieve a higher reversible capacity and good rate performance. However, the traditional strategies for synthesizing N/S co-doped carbon suffer from a series of problems, such as the high cost of raw materials or complicated synthetic routes. It is, therefore, pertinent and worthwhile to further explore low-cost and environmentally-friendly synthetic methods to fabricate N/S co-doped carbon materials for practical applications.

**Scheme 1:**
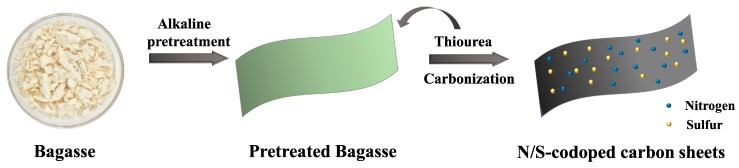
Schematic illustration of the synthesis of N/S-co-doped porous carbon sheets.

Bagasse is a common agricultural waste. Choosing agricultural waste resources as carbon source is important for easing resource consumption and environmental protection. In this work, N/S-co-doped porous carbon sheets (N/S-CS) were prepared by a simple thermal treatment method, using KOH-treated waste bagasse as a carbon source, and thiourea as the N and S precursor. This is illustrated in Scheme 1. KOH treatment has been employed to remove the silicon oxide in bagasse, and chemically activate carbon material to produce pore structures. The resulting N/S-CS materials possess a porous two-dimensional sheet structure, with a thickness of 70–250 nm and a main pore size of 1.8–10 nm. As anode materials for SIBs, the N/S-CS showed excellent sodium storage performances with a high reversible capacity, good rate capability, and stable cycling performance (retaining 155 mA h g^−1^ at 1 A g^−1^ for 2000 cycles).

## 2. Materials and Methods

### 2.1. Synthesis of N/S-Codoped Porous Carbon Sheets

Bagasse was supplied by local market (Hefei, China). All other reactants were purchased from Shanghai Aladdin Biochemical Technology Co. (Shanghai, China). Bagasse was first washed in distilled water, three times, in order to remove impurities, and dried at 100 °C for 12 h. A total of 2 g bagasse was then immersed into 50 mL 1 M KOH solutions at 90 °C for 8 h, and collected by filtration, and dried at 100 °C. The KOH-treated bagasse was immersed in thiourea solution (the mass ratio of bagasse to thiourea is set to be 1:2) and mechanically milled at 300 r min^−1^ for 0.5 h. After drying at 80 °C for 12 h, the as-prepared materials were then calcined at 800 °C in N_2_ for 2 h. The obtained products were then washed with distilled water several times, and dried at 60 °C to obtain the carbonaceous materials, named N/S-CS. The pristine carbon sheets were synthesized, using a similar procedure, without the addition of thiourea, and named CS.

### 2.2. Material Characterization

The surface morphology of the samples were measured by scanning electron microscopy (SEM, Hitachi S-4800, Hitachi Ltd., Tokyo, Japan) equipped with energy-dispersive spectrometer (EDS) analyzer. Transmission electron microscope (TEM) and high-resolution transmission electron microscopy (HRTEM) analysis was performed using a JEOL JEM-2100 TEM (JEOL Ltd., Tokyo, Japan). The phase was checked by X-ray diffractometer (XRD, Rigaku D/max 2500, λ = 1.5406 Å, Rigaku Co., Tokyo, Japan) and Raman spectroscopy (JY-HR 800, Horiba Ltd., Paris, France). The nitrogen adsorption and desorption isotherms, and pore size distributions were characterized by a Micromeritics ASAP 2020 analyzer (Micromeritics Instrument Corp., GA, USA). X-ray photoelectron spectroscopy (XPS) was obtained on an Axis Ultra spectrometer (Kratos Analytical Ltd., Manchester, UK). Elementary analysis (EA) (Elementar vario EL cube, Thermal Conductivity Detector, Elementar GmbH, Hanau, Germany) was used to determine the nitrogen and sulfur content in composites.

### 2.3. Electrochemical Test

The working electrode was prepared by grinding the active materials, Super P and polyvinylidene difluoride (PVDF, 5 wt% in *N*-methyl-2-pyrrolidone) at a weight ratio of 8:1:1. Then, the mixture was coated onto the copper film, and then dried at 110 °C for 10 h in a vacuum oven. CR2025-type half-cells were assembled in a glove box under an argon atmosphere. The electrolyte was comprised of a solution of 1 M NaClO_4_ in ethylene carbonate (EC) and propylene carbonate (PC) mixture (1:1 v/v); glass fibers were used as separators. The cyclic voltammograms (CV) was carried out on an electrochemical workstation (CHI760E, Chenhua Instrument Co. Ltd., Shanghai, China). The galvanostatic charge/discharge measurements were performed using a Land Battery Measurement System (Land, Wuhan, China). The electrochemical impedance spectra (EIS) were performed by using a Zahner IM6 workstation (Zahner Elektrik, Kronach, Germany).

## 3. Results

**Figure 1:**
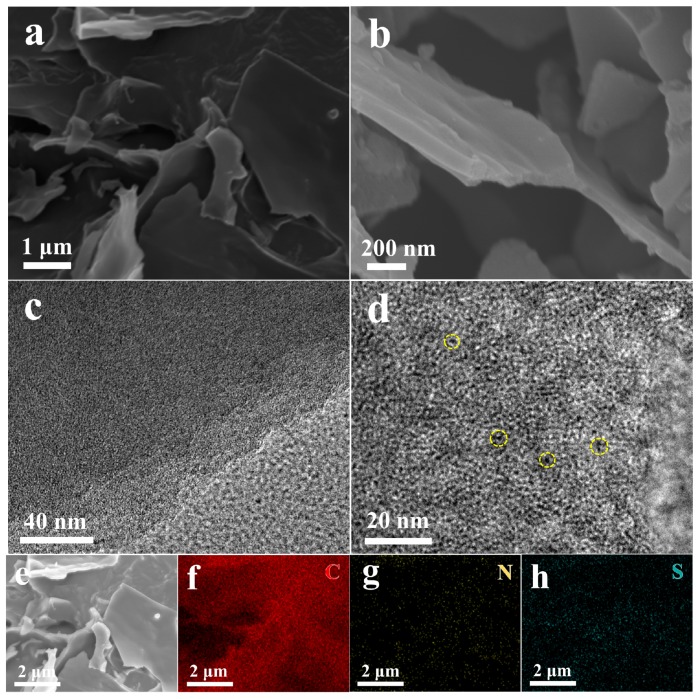
(**a**,**b**) SEM images of N/S-co-doped porous carbon sheets (N/S-CS); (**c**,**d**) TEM and high-resolution transmission electron microscopy (HRTEM) images of N/S-CS; (**e**–**h**) elemental mapping images of N/S-CS.

The morphologies of as-prepared N/S-CS were characterized by SEM. As shown in Figure 1a,b, N/S-CS displays a sheet-like morphology, which inherited the structure of pristine CS (see Figure S1). The thickness of the N/S-CS ranged from 70 to 250 nm. Compared with the micron-sized bulk carbon, the 2D sheet structure had a thinner thickness and was in sufficient contact with the electrolyte to promote good electron/sodium ion transport [16]. The microstructures of N/S-CS were further investigated by TEM and high-resolution transmission electron microscopy (HRTEM). As depicted in Figure 1c,d, no obvious long-range ordered structure can be observed, indicating that N/S-CS has an amorphous structure. The magnified TEM image, in Figure 1 d, clearly shows the presence of abundant nanopores (marked by yellow circle). The average pore size, measured from TEM images, is about 2.1 nm. Such a nano-porous structure will provide a number of channels that allow electrolytes to penetrate throughout the structure and promote Na^+^ transport between the electrode/electrolyte interfaces, as well as to buffer the strain generated during the Na^+^ insertion and extraction process [20]. EDS elemental mapping of N/S-CS (Figure 1e–h) indicates the homogeneous distribution of C, N, and S elements throughout the porous carbon sheets.

**Figure 2:**
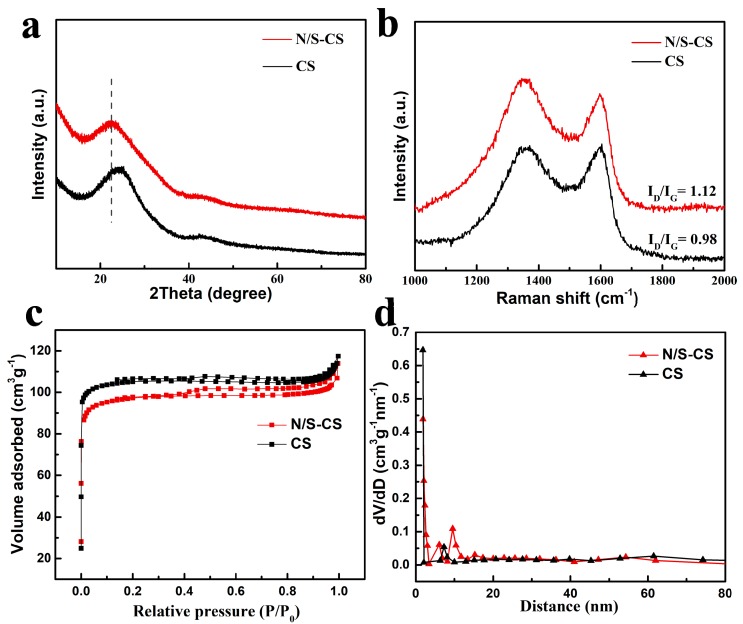
(**a**) XRD patterns of N/S-CS and CS; (**b**) Raman spectra of N/S-CS and CS; (**c**) Nitrogen adsorption/desorption isotherms of N/S-CS and CS; (**d**) Pore size distribution curves of N/S-CS and CS.

Figure 2a displays the XRD patterns of N/S-CS and CS. Two dominant broad peaks of (002) and (100) diffractions can be clearly observed, indicating an amorphous carbon structure of N/S-CS, which is consistent with the HRTEM results. The (002) diffraction peak of N/S-CS located at 22.5°, much lower than that of CS (24.1°). According to Bragg’s equation (2dsinθ = λ, λ = 0.15406 nm), the interlayer distances of N/S-CS is 3.95 Å, which is larger than that of CS (3.69 Å), indicating that the as-obtained N/S-CS should be beneficial for the intercalation/extraction process of Na^+^. Such results show that the introduction of sulfur atoms can enlarge the interlayer distance of disordered carbon, which is coincidence with the previous report [21,24,25]. Since the covalent radius of sulfur is much larger than those of carbon and nitrogen, the substitution will result in an increase in the spacing between adjacent interlayer [26]. Figure 2b displays the Raman spectrum of N/S-CS and CS, and both of them exhibit a characteristic disorder-induced D band (1356 cm^−1^) and interplane vibrational G band (1598 cm^−1^) [15,26]. The relative intensities ratio of the D and G bands (I_D_/I_G_) can be used to quantify the disordered degree of the carbon materials [27]. The N/S-CS exhibits a higher I_D_/I_G_ (1.12) than that of CS (0.98), suggesting increased defect sites within N/S-CS after co-doping, that are consistent with the XRD results. It has been reported that the defect sites, introduced by doping, have lower Na^+^ adsorption energy than the perfect sites, which is beneficial in improving the adsorption stability of Na^+^ [28,29].

To further explore the surface and the porous structures of N/S-CS and CS, N_2_ adsorption-desorption tests were performed. As shown in Figure 2c, both of the two samples show combined characteristics of type I and IV isotherms, indicating the N/S-CS and CS possess hierarchical porous structures, with coexisting mesopores and micropores. The BET specific surface areas of N/S-CS and CS are 367 m^2^ g^−1^, and 378 m^2^ g^−1^, respectively. After N and S atoms doping, the BET specific surface area of NS-CS slightly decreased, compared to the CS. The pore size distribution of the N/S-CS, shown in Figure 2d, is mainly located at 1.8 nm, 6.1 nm, and 9.6 nm, corresponding to the micropores, and mesopores, respectively. Similarly, the pore size distribution of CS is mainly at 1.8 nm and 7.3 nm. Notably, the porous structure and high surface area of the obtained N/S-CS can shorten the diffusion paths for charge carriers in bi-continuous electrode and electrolyte phases, facilitate the mass transfer of Na^+^, and thus can reduce internal resistance and efficiently improve rate capabilities [30,31]. The porous structure and high surface area of the obtained N/S-CS is beneficial in facilitating the fast migration of Na^+^ and in improving the rate performance.

**Figure 3:**
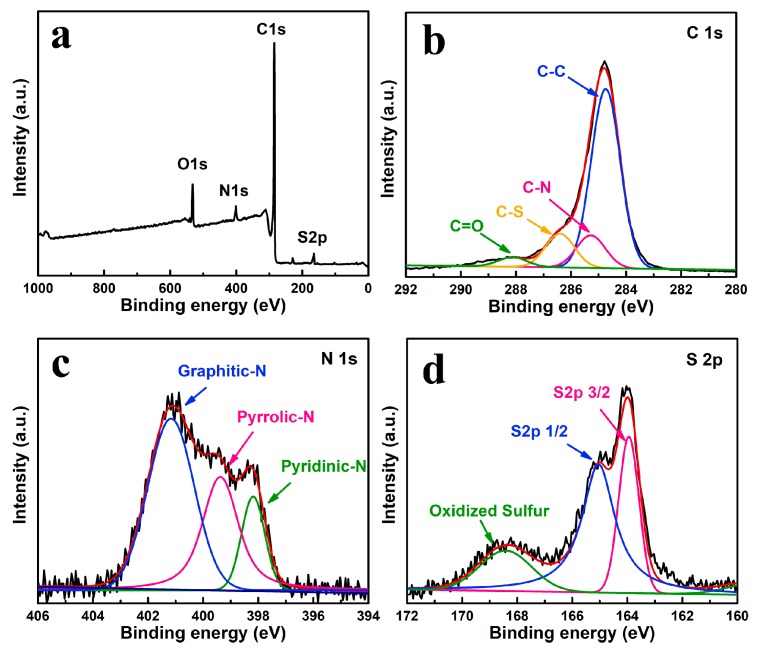
(**a**) XPS survey; (**b**) C 1s; (**c**) S 2p; and (**d**) N 1s spectra of N/S-CS.

XPS was further carried out to investigate the element and chemical bonding configurations of N/S-CS. As shown in Figure 3a, four dominant peaks located at 532, 401, 285, and 165 eV can be detected, corresponding to O 1s, N 1s, C 1s, and S 2p, respectively [32]. All the high-resolution XPS spectra of C 1s (Figure 3b) can be divided into four peaks in different functional groups. The peaks at 284.7, 285.3, 286.4, and 288.1 eV are attributed to the sp^2^ hybridized carbon, C–N bonds, C–S bonds, and C=O bonds, respectively [33,34]. The N 1s spectrum (Figure 3c) of the N/S-CS can be fitted into three components: The graphitic-N (N-Q) at 401.1 eV, pyrrolic-N (N-5) at 399.4 eV, and pyridinic-N (N-6) at 398.2 eV [21]. The graphitic-N and pyridinic-N could enhance the electric conductivity and Na^+^ storage capability of carbon materials [24]. Furthermore, the S 2p spectrum of N/S-CS (Figure 3d) can be deconvoluted into three peaks located at 168.5, 165.0, 163.9 eV. Two main peaks may be assigned to S 2p_3/2_ and S 2p_1/2_. The remaining weak peak at 168.5 eV can be indexed as C–SO_x_–C (x = 2–4) [33,35]. Therefore, these results confirmed that the N and S has been successfully doped into the carbon structure of N/S-CS. While, the N and S content of N/S-CS, which was confirmed by elementary analysis (EA) (Table S1), is approximately 5.93 wt%, and 5.07 wt%, respectively. It is found that the carbonization temperature greatly affects the chemical content of N/S-CS. EA show that the content of N decreases with increasing carbonization temperature. On the contrary, the content of S increases as the carbonization temperature increases, indicating that higher temperatures are required for S doping into the carbon sheet.

**Figure 4:**
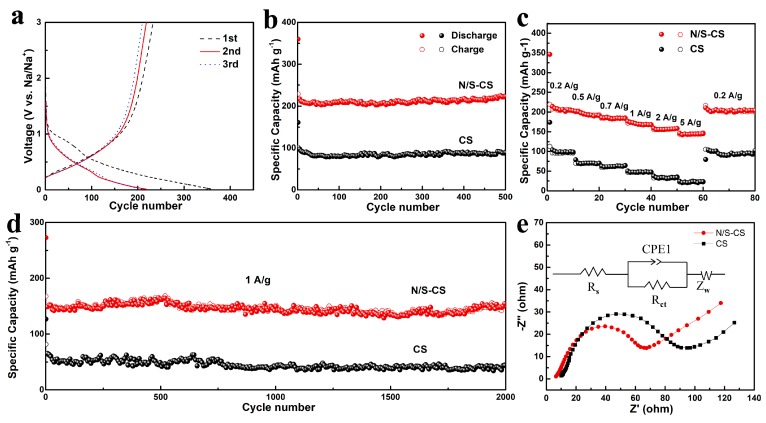
(**a**) Discharge/charge profiles of N/S-CS; (**b**) cycling performances at 0.2 A g^−1^; (**c**) rate performances; and (**d**) long-term cycling performances at 1 A g^−1^ of N/S-CS and CS; (**e**) Nyquist plots of N/S-CS and CS after 200 cycles.

N/S-CS was evaluated as an anode material for sodium ion batteries. The CV curves of N/S-CS and CS samples, measured in the range of 0.01–3.0 V versus Na/Na^+^, at a scan rate of 0.1 mV s^−1^, are shown in Figure S2. For the N/S-CS electrode, two cathodic peaks, at about 0.82 V and 0.47 V, are observed in the first cycle, disappearing in the following cycles, which may be related to the decomposition of the electrolyte and the formation of the solid-electrolyte interphase (SEI) on the carbon surface [36]. At the low voltage region, the N/S-CS electrode exhibits a sharp redox pair, located near 0 V and 0.11 V, corresponding to the Na^+^ insertion into carbonaceous materials, which is similar to Li^+^ insertion into LIBs [15]. There is a broad redox pair over a wide voltage region from 0.15 to 1.2 V, which can be assigned to surface induced capacitive processes at the surface [33]. CV curves of the CS are similar to that of the N/S-CS, indicating a similar mechanism for sodium storage (see Figure S2b). The galvanostatic charge/discharge profiles of N/S-CS electrode at 0.2 A g^−1^, with a cutoff voltage window of 0.01–3.0 V, were studied. As shown in Figure 4a, the first discharge and charge capacities of the N/S-CS are 396 mA h g^−1^, and 233 mA h g^−1^, respectively, corresponding to an initial coulombic efficiency of 58.7%. The large irreversible capacity loss can be ascribed to the inevitable formation of a solid electrolyte interphase (SEI) layer on the relatively large specific surface area and decomposition of electrolytes, which are common to most anode materials [37,38]. The presence of a sloping plateau at around 0.9 V in the first cycle can be attributed to the formation of SEI films [38], which agrees well with the CV results. The theoretical capacity of N/S-CS, based on the N and S content, is calculated to be 370.7 mAh g^−1^ (see Supporting Information). The cycling performance of the N/S-CS and CS electrodes were investigated at 0.2 A g^−1^ (Figure 4b). The N/S-CS electrode exhibits excellent cycling stability and a high reversible capacity of 223 mA h g^−1^ after 500 cycles. While, for the CS electrode, a lower reversible capacity of 88.1 mA h g^−1^ can be obtained, indicating that N and S co-doping can effectively increase the capacity of the electrode. Figure 4c shows the rate performance of the N/S-CS and CS electrodes, with the former showing a much better performance. At current densities of 0.2, 0.5, 0.7, 1, 2, and 5 A g^−1^, stable discharge capacities of N/S-CS were 209, 194, 185, 168, 159, and 148 mA h g^−1^, respectively. When the current density was restored to 0.2 A g^−1^, the capacities well recovered to 206 mA h g^−1^, indicating the excellent rate capability of N/S-CS. A prolonged cycling test was also conducted for N/S-CS and CS electrodes at 1 A g^−1^. As shown in Figure 4d, the specific capacity of N/S-CS electrode can maintain at 155 mA h g^−1^ after 2000 cycles at 1 A g^−1^, suggesting that an extraordinarily stable electrochemical performance among carbonaceous anode materials of SIBs (Table S2, Supporting Information). While, the CS electrode delivers a much lower reversible capacity of 41 mA h g^−1^ after 2000 cycles at the same current density. The coulombic efficiencies of these anodes are stabilized at almost 100% after 5 initial cycles at 1 A g^−1^, indicating their highly reversible nature for efficient Na^+^ storage.

The EIS were further investigated to understand the improved electrochemical performance of the N/S-CS electrodes (Figure 4e). The Nyquist plots of N/S-CS and CS electrodes display one semi-circle, at high-to-medium frequency regions. The diameter of the semi-circular loops correspond to the resistance of charge-transfer resistance (Rct), and the sloped line in the low-frequency region, represents the Warburg impedance [24]. The fitted results of Nyquist plots is shown in Table S3. The Rct of N/S-CS (40.1 Ω) is smaller than that of CS (58.1 Ω), attributed to the increased conductivity and electrochemical activity, are derived from the N and S co-doping.

## 4. Conclusions

In summary, N/S-codoped porous carbon sheets, that have been derived from bagasse, was synthesized via a green and low-cost method. The as-prepared N/S-CS possess a 2D sheet-like porous structure with a thickness of 70–250 nm and a main pore size of 1.8–10 nm, and abundant defects induced by N and S co-doping. Due to the synergistic effect of the 2D porous structure and the co-doping of N and S, N/S-CS exhibits enhanced electron conductivity, shortened diffusion distance of Na^+^, and increased Na^+^ storage sites, and thereby resulting in excellent sodium storage properties. As an anode for SIBs, N/S-CS delivers a high capacity of 223 mA h g^−1^ at 0.2 A g^−1^ after 500 cycles, an excellent rate performance, and a superior long-term cycling performance of 155 mA h g^−1^ at 1 A g^−1^ over 2000 cycles. This work provides a low-cost route to fabricating high-performance dual-doped porous carbonaceous anode materials for SIBs.

## Supplementary Materials

The following are available online at https://www.mdpi.com/2079-4991/9/9/1203/s1, Figure S1: SEM image of CS; Figure S2: Cyclic voltammetry performance of (a) N/S-CS and (b) CS; Table S1: The S and N contents dependence on the annealing temperature; Table S2: Comparison of the electrochemical performance of N/S-CS with other carbon materials reported in previous literature; Table S3: EIS fitting results of the N/S-CS and CS.

## References

[1] Wang L., Lu B., Wang S., Cheng W., Zhao Y., Zhang J., Sun X. (2019). Ultra-high performance of Li/Na ion batteries using N/O dual dopant porous hollow carbon nanocapsules as an anode. J. Mater. Chem. A.

[2] Hu X., Sun X., Yoo S.J., Evanko B., Fan F., Cai S., Zheng C., Hu W., Stucky G.D. (2019). Nitrogen-rich hierarchically porous carbon as a high-rate anode material with ultra-stable cyclability and high capacity for capacitive sodium-ion batteries. Nano Energy.

[3] Kim D.Y., Kim D.H., Kim S.H., Lee E.K., Park S.K., Lee J.W., Yun Y.S., Choi S.Y., Kang J. (2019). Nano hard carbon anodes for sodium-ion batteries. Nanomaterials.

[4] Kim S., Qu S., Zhang R., Braun P.V. (2019). High Volumetric and gravimetric capacity electrodeposited mesostructured Sb_2_O_3_ sodium ion battery anodes. Small.

[5] Liu Y., Fang Y., Zhao Z., Yuan C., Lou X. (2019). Sodium-Ion Batteries: A ternary Fe_1-x_S@porous carbon nanowires/reduced graphene oxide hybrid film electrode with superior volumetric and gravimetric capacities for flexible sodium ion batteries. Adv. Energy Mater..

[6] Zhou Y., Wang Q., Zhu X., Jiang F. (2018). Three-dimensional SnS decorated carbon nano-networks as anode materials for lithium and sodium ion batteries. Nanomaterials.

[7] Zhao D., Zhao R., Dong S., Miao X., Zhang Z., Wang C., Yin L. (2019). Alkali-induced 3D crinkled porous Ti_3_C_2_ MXene architectures coupled with NiCoP bimetallic phosphide nanoparticles as anodes for high-performance sodium-ion batteries. Energy Environ. Sci..

[8] Ma J., Prieto A.L. (2019). Electrodeposition of pure phase SnSb exhibiting high stability as a sodium-ion battery anode. Chem. Commun..

[9] Lei P., Liu K., Wan X., Luo D., Xiang X. (2019). Ultrafast Na intercalation chemistry of Na_2_Ti_3/2_Mn _1/2_(PO_4_)_3_ nanodots planted in a carbon matrix as a low cost anode for aqueous sodium-ion batteries. Chem. Commun..

[10] Luo W., Jian Z., Xing Z., Wang W., Bommier C., Lerner M.M., Ji X. (2015). Electrochemically expandable soft carbon as anodes for Na-ion batteries. ACS Cent. Sci..

[11] Fu L., Tang K., Song K., van Aken P.A., Yu Y., Maier J. (2014). Nitrogen doped porous carbon fibres as anode materials for sodium ion batteries with excellent rate performance. Nanoscale.

[12] Zhang K., Li X., Liang J., Zhu Y., Hu L., Cheng Q., Guo C., Lin N., Qian Y. (2015). Nitrogen-doped porous interconnected double-shelled hollow carbon spheres with high capacity for lithium ion batteries and sodium ion batteries. Electrochim. Acta.

[13] Ye J., Zang J., Tian Z., Zheng M., Dong Q. (2016). Sulfur and nitrogen co-doped hollow carbon spheres for sodium-ion batteries with superior cyclic and rate performance. J. Mater. Chem. A.

[14] Kim N.R., Yun Y.S., Song M.Y., Hong S.J., Kang M., Leal C., Park Y.W., Jin H.J. (2016). Citrus-peel-derived, nanoporous carbon nanosheets containing redox-active heteroatoms for sodium-ion storage. ACS Appl. Mater. Interfaces.

[15] Ding J., Wang H., Li Z., Kohandehghan A., Cui K., Xu Z., Zahiri B., Tan X., Lotfabad E.M., Olsen B.C. (2013). Carbon nanosheet frameworks derived from peat moss as high performance sodium ion battery anodes. ACS Nano.

[16] Yang F., Zhang Z., Du K., Zhao X., Chen W., Lai Y., Li J. (2015). Dopamine derived nitrogen-doped carbon sheets as anode materials for high-performance sodium ion batteries. Carbon.

[17] Wang H.G., Wu Z., Meng F.L., Ma D.L., Huang X.L., Wang L.M., Zhang X.B. (2013). Nitrogen-doped porous carbon nanosheets as low-cost, high-performance anode material for sodium-ion batteries. ChemSusChem.

[18] Song H., Li N., Cui H., Wang C. (2014). Enhanced storage capability and kinetic processes by pores-and hetero-atoms-riched carbon nanobubbles for lithium-ion and sodium-ion batteries anodes. Nano Energy.

[19] Wang X., Li G., Hassan F.M., Li J., Fan X., Batmaz R., Xiao X., Chen Z. (2015). Sulfur covalently bonded graphene with large capacity and high rate for high-performance sodium-ion batteries anodes. Nano Energy.

[20] Li W., Zhou M., Li H., Wang K., Cheng S., Jiang K. (2015). A high performance sulfur-doped disordered carbon anode for sodium ion batteries. Energy Environ. Sci..

[21] Ye J., Zhao H., Song W., Wang N., Kang M., Li Z. (2019). Enhanced electronic conductivity and sodium-ion adsorption in N/S co-doped ordered mesoporous carbon for high-performance sodium-ion battery anode. J. Power Sources.

[22] Shi X., Chen Y., Lai Y., Zhang K., Li J., Zhang Z. (2017). Metal organic frameworks templated sulfur-doped mesoporous carbons as anode materials for advanced sodium ion batteries. Carbon.

[23] Yang J., Zhou X., Wu D., Zhao X., Zhou Z. (2017). S-Doped N-Rich Carbon nanosheets with expanded interlayer distance as anode materials for sodium-ion batteries. Adv. Mater..

[24] Qie L., Chen W., Xiong X., Hu C., Zou F., Hu P., Huang Y. (2015). Sulfur-doped carbon with enlarged interlayer distance as a high-performance anode material for sodium-ion batteries. Adv. Sci..

[25] Xu D., Chen C., Xie J., Zhang B., Miao L., Cai J., Huang Y., Zhang L. (2016). A hierarchical N/S-codoped carbon anode fabricated facilely from cellulose/polyaniline microspheres for high-performance sodium-ion batteries. Adv. Energy Mater..

[26] Wan H., Hu X. (2019). Nitrogen/sulfur co-doped disordered porous biocarbon as high performance anode materials of lithium/sodium ion batteries. Int. J. Hydrog. Energy.

[27] Ruan J., Yuan T., Pang Y., Luo S., Peng C., Yang J., Zheng S. (2018). Nitrogen and sulfur dual-doped carbon films as flexible free-standing anodes for Li-ion and Na-ion batteries. Carbon.

[28] Yang C., Xiong J., Ou X., Wu C.F., Xiong X., Wang J.H., Huang K., Liu M. (2018). A renewable natural cotton derived and nitrogen/sulfur co-doped carbon as a high-performance sodium ion battery anode. Mater. Today Energy.

[29] Datta D., Li J., Shenoy V.B. (2014). Defective graphene as a high-capacity anode material for Na-and Ca-ion batteries. ACS Appl. Mater. Interfaces.

[30] Yue X., Huang N., Jiang Z., Tian X., Wang Z., Hao X., Jiang Z.J. (2018). Nitrogen-rich graphene hollow microspheres as anode materials for sodium-ion batteries with super-high cycling and rate performance. Carbon.

[31] Hou H., Shao L., Zhang Y., Zou G., Chen J., Ji X. (2017). Large-area carbon nanosheets doped with phosphorus: A high-performance anode material for sodium-ion batteries. Adv. Sci..

[32] Qiu Z., Lin Y., Xin H., Han P., Li D., Yang B., Li P., Ullah S., Fan H., Zhu C. (2018). Ultrahigh level nitrogen/sulfur co-doped carbon as high performance anode materials for lithium-ion batteries. Carbon.

[33] Zou G., Hou H., Zhao G., Huang Z., Ge P., Ji X. (2017). Preparation of S/N-codoped carbon nanosheets with tunable interlayer distance for high-rate sodium-ion batteries. Green Chem..

[34] Cazetta A.L., Martins A.C., Pezoti O., Bedin K.C., Beltrame K.K., Asefa T., Almeida V.C. (2016). Synthesis and application of N-S-doped mesoporous carbon obtained from nanocasting method using bone char as heteroatom precursor and template. Chem. Eng. J..

[35] Luan D., Wu L., Wei T., Liu L., Lv Y., Yu F., Chen L., Shi Y. (2019). N, S Dual-doped carbon derived from dye sludge by using polymeric flocculant as soft template. Nanomaterials.

[36] Cao Y., Xiao L., Sushko M.L., Wang W., Schwenzer B., Xiao J., Nie Z., Saraf L.V., Yang Z., Liu J. (2012). Sodium ion insertion in hollow carbon nanowires for battery applications. Nano Lett..

[37] Xiong J., Pan Q., Zheng F., Xiong X., Yang C., Hu D., Huang C. (2018). N/S Co-doped carbon derived from cotton as high performance anode materials for lithium ion batteries. Front. Chem..

[38] Cao X., Yang Y., Li A. (2018). Facile synthesis of porous ZnCo_2_O_4_ nanosheets and the superior electrochemical properties for sodium ion batteries. Nanomaterials.

