# Supplementary material for: N/S-Co-Doped Porous Carbon Sheets Derived from Bagasse as High-Performance Anode Materials for Sodium-Ion Batteries
Source: Nanomaterials (Basel). 2019 Aug 27;9(9):1203. doi: 10.3390/nano9091203 (PMC6781196; doi:10.3390/nano9091203)
Supplement: Supplementary file 1 [file nanomaterials-09-01203-s001.pdf]

Supplementary Materials

N/S-Co-Doped Porous Carbon Sheets Derived from Bagasse as High-Performance Anode Materials for Sodium-Ion Batteries

Lili Wang *, Lei Hu, Wei Yang, Dewei Liang, Lingli Liu, Sheng Liang, Caoyu Yang, Zezhong Fang, Qiang Dong and Chonghai Deng *

Department of Chemical and Materials Engineering, Hefei University, Hefei 230601, China

* Correspondence: wangll@hfuu.edu.cn; (L.W.); chdeng@mail.ustc.edu.cn (C.D.)

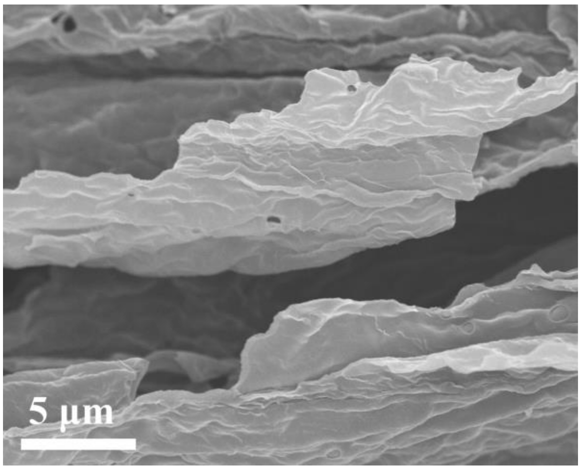

Figure S1. SEM image of CS.

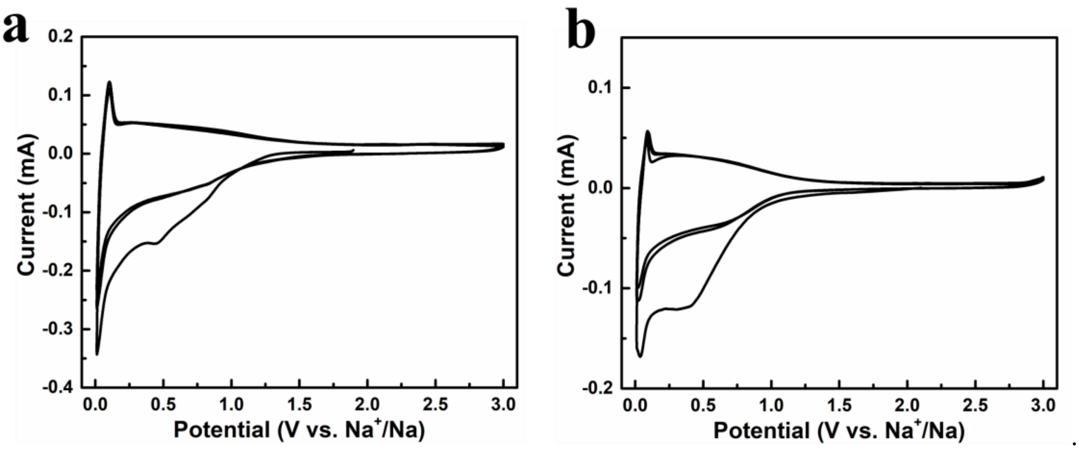

Figure S2. Cyclic voltammetry performance of (a) N/S-CS and (b) CS.

Calculation method of the theoretical capacity of N/S-CS:

The calculation formula of theoretical capacity is $C = \frac{96500*n}{3.6}$. According to calculation method of previous work (*J. Am. Chem. Soc.* **2012**, 134, 4505-4508), the theoretical capacity of N/S-CS is as follows:

$C_{\text{theoretical capacity}} =$ 0.0593*C_N+0.0507*C_s+0.89*C_c (C_N= 638 mAh g^{-1}, C_S= 1675 mAh g^{-1}, C_c= 279 mAh g^{-1})

Therefore, $C_{\text{theoretical capacity}}$ =37.8 + 84.9 + 248 = 370.7 mAh g^{-1}.

Table S1. The S and N contents dependence on the annealing temperature.

Carbonization temperature (°C)	N content (wt%)	S content (wt%)
900	4.24	6.09
800	5.93	5.07
700	7.37	3.89

Table S2. Comparison of the electrochemical performance of N/S-CS with other carbon materials reported in previous literature.

Carbon materials	Precursor	Capacity and cycle stability	Ref.
Carbon nanosheet frameworks	Peat moss	255 mA h g^{-1} after 210 cycles at 0.1 A g^{-1}	1
Nanoporous carbon nanosheets	Citrus peel	53 mA h g^{-1} after 2000 cycles at 1 A g^{-1}	2
N-doped porous carbon fiber	Polypyrrole	72 mA h g^{-1} after 100 cycles at 10 A g^{-1}	3
S-Doped carbon	Poly(3,4-ethylenedioxythiophen e)	303 mA h g^{-1} after 700 cycles at 0.5 A g^{-1}	4
N-doped carbon sheets	Graphene	88.9 mA h g^{-1} after 260 cycles at 1 A g^{-1}	5
N/O-codoped carbon	1-alkyl-3-methylimidazolium bromide	60 mA h g^{-1} after 30 cycles at 0.2 A g^{-1}	6
S/N/O-tridoped porous carbons	Carrageen	157 mA h g^{-1} after 500 cycles at 1 A g^{-1}	7
N/S co-doped carbon	Gelatin	300 mA h g^{-1} after 500 cycles at 0.2 A g^{-1}	8
S/N-co-doped hollow carbon spheres	Polymethyl methacrylate	169 mA h g^{-1} after 2000 cycles at 0.5 A g^{-1}	9
N/S-CS	Bagasse	155 mA h g^{-1} after 2000 cycles at 1 A g^{-1}	This work

Table S3. EIS fitting results of the N/S-CS and CS.

Sample	R_s/Ω	R_{ct}/Ω
N/S-CS	4.3	40.1
CS	7.1	58.1

References

1. Ding, J.; Wang, H.; Li, Z.; Kohandehghan, A.; Cui, K.; Xu, Z.; Zahiri, B.; Tan, X.; Lotfabad, E.M.; Olsen, B.C.; et al. Carbon nanosheet frameworks derived from peat moss as high performance sodium ion battery anodes. *ACS Nano* **2013**, 7 (12) and 11004–11015.
2. Kim, N.R.; Yun, Y.S.; Song, M.Y.; Hong, S.J.; Kang, M.; Leal, C.; Park, Y.W.; Jin, H.J.; Citrus-peel-derived, nanoporous carbon nanosheets containing redox-active heteroatoms for sodium-ion storage. *ACS Appl. Mater. Inter.* **2016**, 8 (5), 3175–3181.
3. Fu, L.; Tang, K.; Song, K.; van Aken, P.A.; Yu, Y.; Maier, J.; Nitrogen doped porous carbon fibres as anode materials for sodium ion batteries with excellent rate performance. *Nanoscale* **2014**, 6 (3), 1384-1389.
4. Qie, L.; Chen, W.; Xiong, X.; Hu, C.; Zou, F.; Hu, P.; Huang, Y.; Sulfur-Doped Carbon with Enlarged Interlayer Distance as a High-Performance Anode Material for Sodium-Ion Batteries. *Adv. Sci.* **2015**, 2 (12), 1500195.
5. Wang, H. g.; Wu, Z.; Meng, F. l.; Ma, D. l.; Huang, X. l.; Wang, L. m.; Zhang, X. b., Nitrogen-doped porous carbon nanosheets as low-cost, high-performance anode material for sodium-ion batteries. *ChemSusChem* **2013**, 6 (1), 56–60.
6. Song, H.; Li, N.; Cui, H.; Wang, C.; Enhanced storage capability and kinetic processes by pores-and hetero-atoms-riched carbon nanobubbles for lithium-ion and sodium-ion batteries anodes. *Nano Energy* **2014**, *4*, 81–87.
7. Lu, M.; Yu, W.; Shi, J.; Liu, W.; Chen, S.; Wang, X.; Wang, H.. Self-doped carbon architectures with heteroatoms containing nitrogen, oxygen and sulfur as high-performance anodes for lithium-and sodium-ion batteries. *Electrochim. Acta* **2017**, *251*, 396–406.
8. Qiao, Y.; Ma, M.; Liu, Y.; Li, S.; Lu, Z.; Yue, H.; Dong, H.; Cao, Z.; Yin, Y.; Yang, S.. First-principles and experimental study of nitrogen/sulfur co-doped carbon nanosheets as anodes for rechargeable sodium ion batteries. *J. Mater. Chem. A* **2016,** *4* (40), 15565–15574.
9. Ye, J.; Zang, J.; Tian, Z.; Zheng, M.; Dong, Q.. Sulfur and nitrogen co-doped hollow carbon spheres for sodium-ion batteries with superior cyclic and rate performance. *J. Mater. Chem. A* **2016,** *4* (34), 13223–13227.
